# Case report: The management of advanced oral cancer in a Jehovah's Witness using the Ultracision Harmonic Scalpel

**DOI:** 10.1186/1477-7819-9-115

**Published:** 2011-10-03

**Authors:** Peter J Kullar, Kristian Sorenson, Ruwan Weerakkody, James Adams

**Affiliations:** 1 Department of Maxillofacial Surgery Royal Victoria Hospital Newcastle-Upon-Tyne UK; 2 Department of Plastic Surgery Royal Victoria Hospital Newcastle-Upon-Tyne UK

**Keywords:** Harmonic scalpel, Head and neck cancer, Jehovah's Witness

## Abstract

We present the first case of a head and neck oncological procedure accomplished in a Jehovah's Witness using the Ultracision Harmonic Scalpel (Ethicon, Cincinnati, OH). Jehovah's Witnesses present a serious challenge to the head and neck cancer surgeon due to their refusal to accept transfusion of any blood products. However, our experience reinforces the view that surgical management of head and neck cancer is possible in these patients. We show the Harmonic Scalpel, an ultrasonic tissue dissector, to be a useful surgical tool in obviating the need for blood transfusion. Preoperative optimisation, intra-operative surgical and anaesthetic techniques are also fully discussed.

## Background

**Figure 1:**
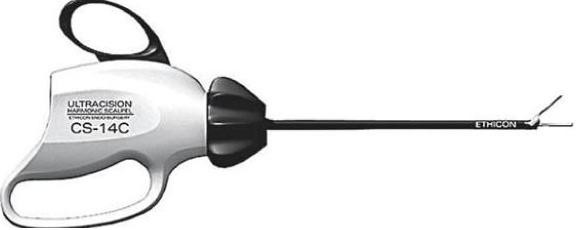
**Ultracision Harmonic Scalpel (Ethicon, Cincinnati, OH)**. http://www.ethiconendosurgery.com/Clinician/Product/energy/.

Jehovah's Witnesses (JW) are a substantial Christian denomination, numbering up to 7 million with a presence in almost all countries worldwide. They are governed by a group of elders exercising authority on all doctrinal matters based on their own translation of the bible. Of particular relevance to medical practice is their refusal, since 1945, to accept blood transfusions even in cases of medical emergency [1] This has been the centre of a number of high profile medical ethics cases [2]. The Harmonic Scalpel (HS), an ultrasonic dissector coagulator (Figure 1; Ethicon, Cincinnati, OH), is a new surgical tool which simultaneous cuts and coagulates tissues. Here we report a case of a large oral cancer and neck dissection with free flap reconstruction performed in a JW with the HS obviating the need for blood products.

## Case presentation

A 48 year old female Caucasian JW presented with non-healing ulcerated lower right second and third molar extraction sockets in 2005. Her past medical history was unremarkable. She was a non-smoker and consumed only moderate amounts of alcohol in accordance with her religious beliefs. Biopsy was performed confirming a moderately differentiated squamous cell carcinoma (SCC), staged by whole body computed tomography (CT) as T4N0M0. She was initially treated with primary chemoradiotherapy as blood products were deemed to be essential for surgical resection at this time. She received 63 Grays of radiation and 5 cycles of cisplatin. Post-treatment she was deemed to be tumor free.

In 2006 she presented with an unstable ulcer on the left lateral tongue. Biopsy revealed early invasive pT1 SCC. This was treated with laser excision.

**Figure 2:**
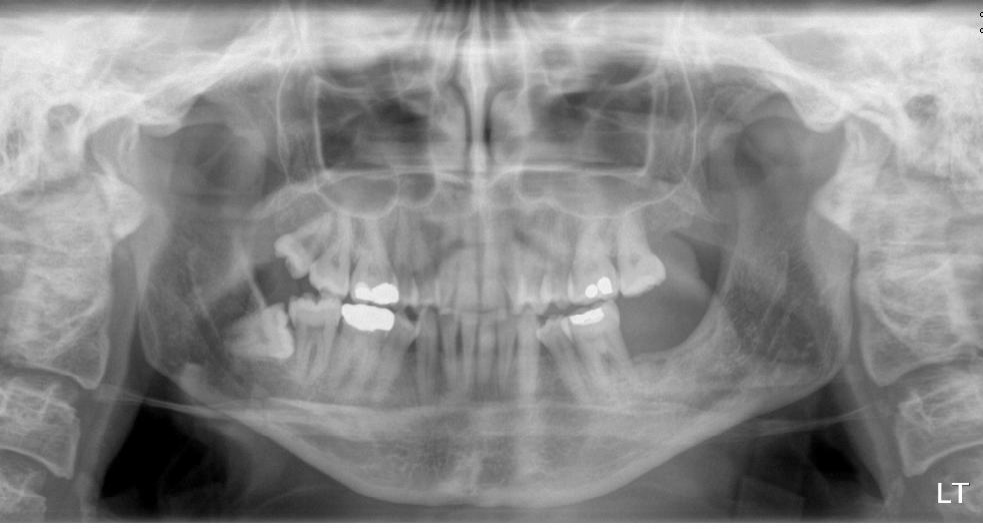
**Panorex image demonstrating lower right retromolar mass eroding into the mandible (2009)**.

She was followed up at monthly intervals, however, in 2009 she presented with a lower right retromolar mass eroding into the mandible (Figure 2, Panorex image). Biopsy revealed Human Papiloma Virus (HPV) negative SCC staged at CT as T4N0M0. Treatment plans were devised after extensive discussion at the Head and Neck Cancer Multi-Disciplinary Team (MDT) meeting. This case was particularly complex given the previous chemoradiotherapy and the inability to use blood products in the case of severe blood loss. Salvage surgery was deemed most appropriate with the proviso that new surgical techniques could now minimize blood loss.

As the patient was unwilling to accept any blood products all efforts were directed at preoperatively optimisation and minimization of intra-operative blood loss. Preoperative blood tests showed haemoglobin (Hb) of 11 g/dl, platelets 310 × 10^9^/l and a normal clotting profile. She was assessed by a haematologist who prescribed a 2 week preoperative course of oral ferrous sulphate. No further preoperative treatment was instigated.

At operation the patient was premedicated with a *statim *dose of 1 gram tranexamic acid and anaestethseia was induced with a combination of propofol (120 mg), fentanyl (100 mcg) and vecuronium (8 mg). Anaesthesia was maintained using a remifentanil infusion (50 mcg/ml at 10 ml/hr) and sevofluorane gas inhalation. Hypervolameic hemodilution was performed using preloading with 15 ml/kg cryastalloid fluid in conjunction with the controlled hypotension using remifentanil infusion maintaining a mean arterial pressure of 65 mmHg. The patient was placed in a head up position and meticulous attention to blood loss was performed throughout the whole operation. Blood loss was charted continuously during the operation. Intra-operative blood sampling also provided a guide to blood loss and the Hb did not drop below 9.6 g/dl during the operation. The intraoperative clotting profile revealed a normal prothrombin (PT) and activated partial throboplastin time (APTT).

**Figure 3:**
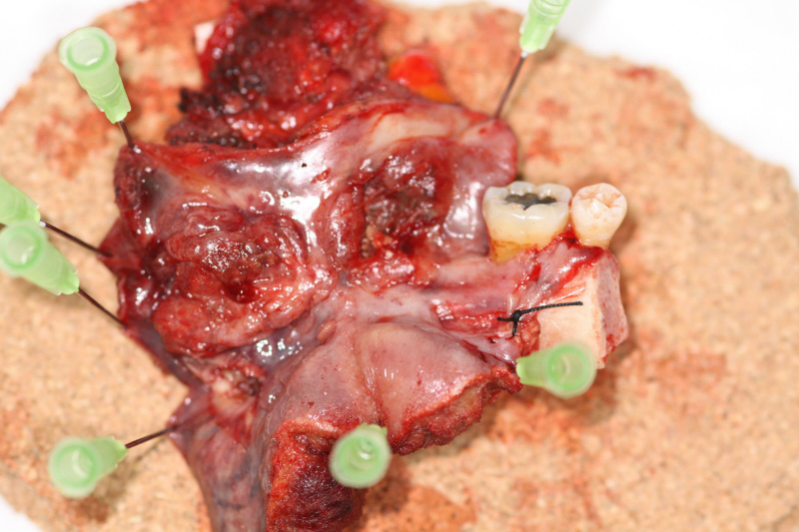
**Left tonsillar and retromolar with mandibular rim resection specimen marked with surgical pins to allow orientation by histopathologist**.

**Figure 4:**
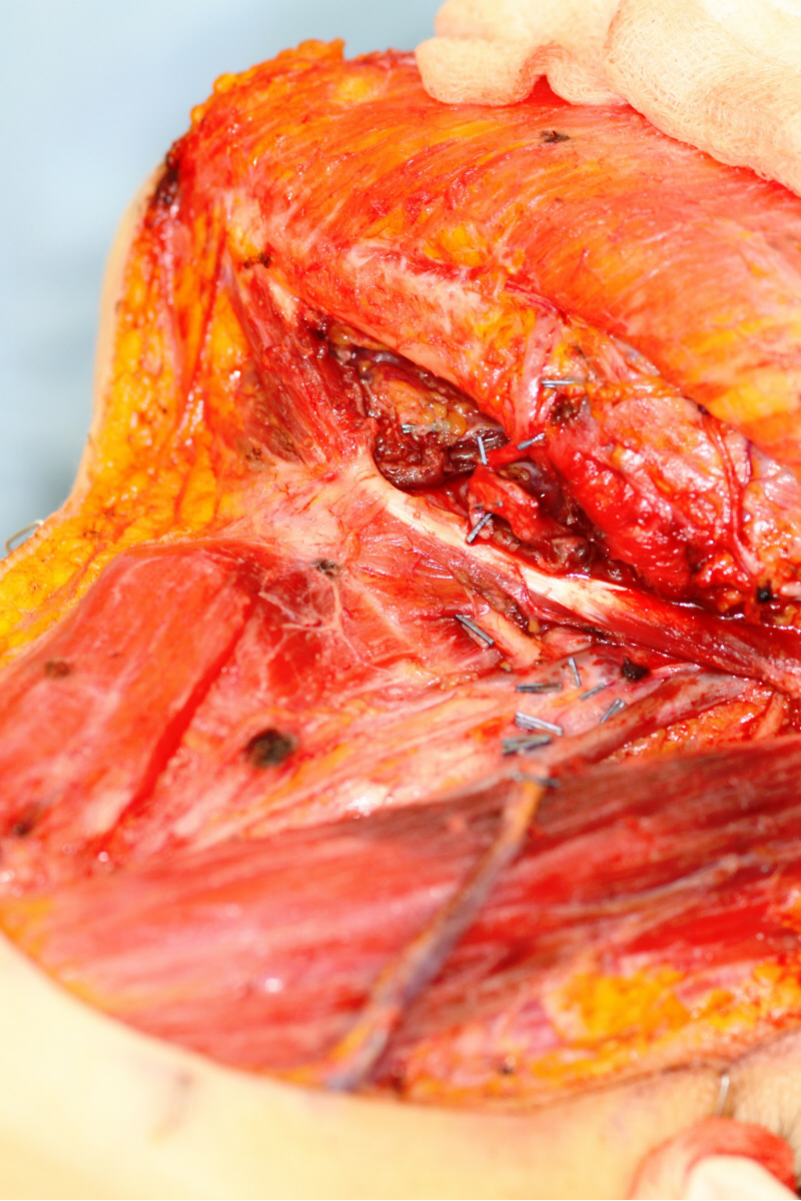
**Intra-operative image demonstrating a left level I-IV neck dissection performed with the Harmonic Scalpel**.

A left tonsillar and retromolar with mandibular rim resection was undertaken and reconstructed with a radial forearm free flap (Figure 3, resection specimen). A left level I-IV neck dissection and covering tracheostomy was also performed (Figure 4, neck dissection). The surgeon (JA) used the HS throughout the procedure. The radial forearm flap was raised by the Plastics Surgical team (KS) using a standard technique with cold steel dissection.

The entire procedure was accomplished in approximately 11 hours. The total blood loss was under 500 ml. During the operation Hartmann's solution was infused at 100 ml/hr (Total 1.1 L). The urine output was 500 ml giving an intra-operative fluid balance of +0.1 L.

The patient was transferred to the intensive care unit for close monitoring on the first postoperative night. She was then returned to the specialised otolaryngology ward. The patient had a single episode of self resolving supraventricular tachycardia but the rest of the postoperative course was uneventful. She was discharged home able to eat a soft diet and with good speech function 2 weeks postoperatively. Her postoperative haemoglobin did not drop below 10 g/dl on her daily postoperative blood counts. No blood products were used at any time.

## Discussion

The use of a blood transfusion is forbidden under JW doctrine. Doctors have no legal right to transfuse someone against their will even if this may result in their death [3]. After extensive discussion at the MDT meeting and with the patient, a comprehensive literature review was undertaken by the operating surgeons. In conjunction with their previous experience of decreased blood loss whilst using the HS it was decided that salvage surgery was the most appropriate course of action.

Blood transfusion, although potentially life-saving is known to be associated with a number of risks. These must be balanced against the potential benefit of transfusion. Reactions range from the self-limiting *febrile non-hemolytic transfusion reaction *to life threatening conditions such as *Transfusion Related Acute Lung Injury *(TRALI)[4]. The transmission of blood borne bacteria and viruses (including HIV and hepatitis B/C) also remains a possibility [5,6]. Specifically in surgical patients there is a documented link between transfusions and postoperative infections and tumor recurrence [7].

According to the literature, blood transfusion is required in 14-80% of all head and neck cancer operations [8]. However, within our unit only 16% of major head and neck operations required blood transfusion (11 of 68 cases transfused between August 2008-August 2009). We have also noted a significant decline in the use of transfusion since we began to use the HS (unpublished data).

In the preoperative and intra-operative periods hypervolameic/normovolemic hemodiltion [9], hypotensive anaesthesia [10] and cell scavenging [11] have all been described to minimize blood loss. Acute normovolemic hemodilution is the process of removing one or more units of blood before the operation for transfusion to the patient either during or at the end of the operation. This was not acceptable to our patient as blood cannot be reused once taken out of circulation. On discussion with our patient cell scavenging was also ruled out as it involves the reinfusion of blood which has left the circulation. We decided on a strategy of hypervolemic hemodilution. Here considerable fluid is administered intravenously to the patient reducing the patient's hematocrit and hence the blood's oxygen carrying capability. This reduces the number of red blood cells lost intra-operatively. The hematocrit is restored with a postoperative diuresis. This strategy is not suitable for patients with cardiovascular or renal dysfunction [9]. We also used controlled hypotension with a mean arterial pressure of 65 mmHg maintained with an opiate infusion. Similarly, the use of controlled hypotension is only recommended in patients known to be free of cardiovascular and respiratory problems [10].

The use of aprotinin and tranxemic acid have also been employed to reduce intra-operative bleeding [12]. Previous surgery in JW have used aprotinin, a bovine derived inhibitor of trypsin and plasmin. However, this was withdrawn in May 2008 due an increased risk of death in cardiac surgical patients [13]. Therefore we followed the recommendation of Henry et al to use the antifibrinolytic agent tranxemic acid. Their meta-analysis showed this to be the most effective antifibinolytic since the withdrawl of aprotinin [12].

There are only a few documented cases of JWs undergoing major head and neck surgery previously presented in the literature. Genden et al present the case of a 35 year old male with a T3N0M0 anterior floor of mouth SCC who successfully underwent a segmented mandibulectomy and staged fibula free flap reconstruction with osseointegrated dental implants without use of allogenic blood [14]. Van Hemelen et al present two JWs with oral cancer requiring neck dissection and free flap transfer without use of blood transfusion [15]. Skoner et al's presentation of 5 JWs with head and neck cancers requiring 7 free flap reconstructions is the largest series in the literature so far [16]. However, our case is the first presentation of the use of the HS in this patient population.

Preoperative planning requires extensive discussion between all parties involved. Particularly, the patient must be aware that refusal of blood products may have life endangering consequences. Our patient was fully optimized before surgery. A full medical and surgical history was taken to identify any risk factors adversely affecting hemostasis. We restricted pre- and post-operative phlebotomy to a minimum. Preoperative haemoglobin was optimized by a course of ferrous sulphate. We considered the use of recombinant erythropoientin (EPO), to raise preoperative haemoglobin levels. EPO is a glycosylated polypeptide released by the kidney in response to hypoxia which increases erythropoietic precursors in the bone marrow and has been shown to be a useful in avoiding transfusion in surgical specialties including head and neck [17,18]. Remmers et al suggested the use of EPO and iron 2 to 3 weeks before elective surgery in JW [19].

However, the use of EPO has been associated with increased recurrence in head and neck cancer patients [20]. Janecka speculates that this may be due to EPO promoting thrombogenesis and hence tumor hypoxia and angiogenesis [21]. Conversely, a more recent trial failed to shown any negative impact of EPO therapy on patient survival or tumor recurrence [22]. We decided against the use of EPO in this case. The use of recombinant clotting factors and DDAVP was also thought inappropriate in a patient with a physiologically normal clotting profile.

With our unit's experience of the HS and a comprehensive literature review we felt the use of the HS would allow meticulous hemostatis and thus reduce the chances of requiring blood products. The traditional surgical method for head and neck cancer resections is 'cold steel' dissection. Here blood loss is controlled using pressure or ligatures. Monopolar and bipolar electrosurgery are newer methods of dissection which use heat to cauterise bleeding vessels. The HS is a surgical cutting device which concurrently coagulates tissues. The basic principle of this device is converting an ultrasonic wave into high frequency mechanical energy, vibrating the cutting surfaces at 55.5 Khz. The cutting blade is able to denature proteins into a coagulum which acts as a haemostatic seal around blood vessels [23]. This allows optimal surgical visibility in a near bloodless operating field [24]. The harmonic scalpel operates at lower temperatures (50-100 C) compared to mono- or bipolar devices (100-600 C) hence there is less tissue damage and the subsequent inflammatory response is also lessened. The lower operating temperatures mean that neighbouring structures are at less risk of damage hence allowing the surgeon to work around delicate structures [25].

There is also less eschar formation and hence less smoke production. Due to the lower temperatures of the harmonic scalpel as compared to conventional thermal surgical instruments less tissue sticks to the blades. This decreases tissue damage and wasted time cleaning and exchanging instruments [26,27].

A number of studies have shown that operative time, blood loss and hospital stay are shortened in a wide range of head and neck such as tonsillectomy [28] and thyroidectomy [25-27], general [29] and gynaecological surgery [30]. Initial results have been favourable with less postoperative pain, lower blood loss, shortened operative time and lack of impairment of histopathologic evaluation.

Salami et al present a non-randomized study comparing 40 pharyngolarygectomy, 40 total laryngectomy, 40 unilateral neck dissections and 40 superficial parotidectomies with either the harmonic scalpel or 'cold steel' dissection [31]. The evaluation demonstrated a significant decrease in operative time, intra-operative blood loss, total hospital stay, postoperative seroma formation and subjective pain scores for patients treated with the HS.

## Conclusions

In summary, JW present a major challenge to the head and neck cancer surgeon. We believe preoperative optimisation with ferrous sulphate, the use of hypervolameic hemodilution with the addition of tranexamic acid and meticulous attention to intra-operative hemostasis with the HS are the cornerstones of management for these patients. We found the use of the HS in conjunction with our anaesthetic techniques created an almost bloodless operating field and enabled a large cancer resection and reconstruction to be performed without recourse to blood transfusion. We feel the HS will play an important role in bloodless surgery in the future.

## Consent

Written informed consent was obtained from the patient's next of kin for publication of this case report and accompanying images. A copy of the written consent is available for review by the Editor-in-Chief of this journal.

## Competing interests

The authors declare that they have no competing interests.

## Authors' contributions

PK and KS prepared the manuscript. JA was lead surgeon and reviewed the manuscript. All authors read and approved the final version.
